# Supplementary material for: Accelerated polymerization of N-carboxyanhydrides catalyzed by crown ether
Source: Nat Commun. 2021 Feb 2;12:732. doi: 10.1038/s41467-020-20724-w (PMC7854670; doi:10.1038/s41467-020-20724-w)
Supplement: Supplementary file 1 — Supplementary Information [file 41467_2020_20724_MOESM1_ESM.pdf]

Supplementary Information

Accelerated Polymerization of *N*-carboxyanhydrides Catalyzed by Crown Ether

Xia et al.

Supplementary Methods

Materials

All reagents and solvents were obtained from MilliporeSigma (St. Louis, MO, USA) and used without purification unless otherwise specified. Amino acids were purchased from Chem-Impex (Wood Dale, IL, USA). 24-Crown-8 was purchased from TCI America (Portland, OR, USA). Deuterated solvents were purchased from Cambridge Isotope Laboratories, Inc. (Tewksbury, MA, USA). Anhydrous dichloromethane (DCM) and chloroform were stored over 4Å sieves in a freezer. Dry hexanes and tetrahydrofuran (THF) were dried by activated alumina columns. γ-Benzyl-L-glutamate *N*-carboxyanhydride (BLG-NCA), γ-ethyl-L-glutamate *N*-carboxyanhydride (ELG-NCA), N^{ε}-carboxybenzyl-L-lysine *N*-carboxyanhydride (ZLL-NCA), and γ-(4-propargyloxy)benzyl-L-glutamate-*N*-carboxyanhydride (POB-NCA) were synthesized according to previous literatures[1, 2]. Helical poly(γ-benzyl-L-glutamate) (PBLG) bearing an amine terminus (PBLG-NH_2) or an acetyl-capped terminus (PBLG-NHAc) was prepared according to literature procedures[3]. Briefly, PBLG-NH_2 was prepared by polymerizing BLG-NCA at 4 °C in DMF with *n*-hexylamine (Hex-NH_2) as the initiator ($[M]_0/[I]_0 = 30$). The resulting PBLG was purified by precipitation in cold hexane/ether (1:1, v/v) and stored at -30 °C. The low temperature during synthesis and storage minimizes the loss of amino groups at the chain terminus[3, 4]. Pyrenyl- and propargyl-functionalized macroinitiators were prepared in a similar way. Gel permeation chromatography (GPC) and matrix-assisted laser desorption ionization time-of-flight (MALDI-TOF) MS characterization revealed well-defined polypeptides (see Supplementary Fig. 6a, 6b, and 7a) with well-reserved end groups. PBLG-NHAc was prepared by reacting PBLG-NH_2 with acetic anhydride (40 equiv.) at room temperature in DMF overnight, which was purified by precipitation in cold ether.

Instrumentation

Nuclear magnetic resonance (NMR) spectra were recorded on a Varian VXR500, U500 or VNS750NB spectrometer in the NMR laboratory, University of Illinois. Chemical shifts were

reported in ppm and the residual protons in the deuterated solvents were used as internal reference. Fourier transform infrared (FTIR) spectra were operated on a Spectrum 100 spectrometer (Perkin Elmer, Santa Clara, CA, USA) in a SL-3 Model 0.1 mm KBr sealed liquid cell (International Crystal Laboratories, Garfield, NJ, USA). GPC was performed on a system equipped with an isocratic pump (1260 Infinity II, Agilent, Santa Clara, CA, USA), a multi-angle static light scattering (MALS) detector with a 658 nm light source (DAWN HELEOS-II, Wyatt Technology, Santa Barbara, CA, USA), and a differential refractometer (DRI) detector operating at a wavelength of 658 nm (Optilab T-rEX, Wyatt Technology, Santa Barbara, CA, USA). Separations were performed using serially connected size exclusion columns (three PLgel MIXED-B columns, 10 μm, 7.5 × 300 mm, Agilent, Santa Clara, CA, USA) at 40 °C, with DMF containing 0.1 M LiBr as a mobile phase at a flow rate of 0.7 mL/min. Samples were filtered using a 0.45 μm PTFE filter before injection. Absolute molecular weights (MWs) of polymers were determined using the ASTRA 7 software (version 7.1.3, Wyatt Technology, Santa Barbara, CA, USA) and calculated from d*n*/d*c* values of each polymer sample. Circular dichroism (CD) measurements were carried out on a JASCO J-815 CD spectrometer (JASCO, Easton, MD, USA). High performance liquid chromatography (HPLC) was performed on a LC system (Shimadzu, LC-20AT, Columbia, MD, USA) with PDA detector (SPD-M20A) and an analytical C18 column (Eclipse plus, 3.5 μm, 4.6 × 100 mm, Agilent, Santa Clara, CA, USA). Electrospray ionization (ESI) and MALDI-TOF mass spectra were collected in the mass laboratory, University of Illinois. ESI spectra were collected from Waters Synapt G2-Si mass spectrometer (Waters, Milford, MA, USA). MALDI-TOF spectra were obtained on a Bruker ultrafleXtreme (Bruker, Billerica, MA, USA) with α-cyano-4-hydroxycinnamic acid (CHCA) as the matrix.

CE-catalyzed polymerization of NCAs

Crown ether (CE)-catalyzed polymerization of NCA was carried out at room temperature. Typically, the DCM solution of 18-crown-6 (18-C-6, 0.01 M, 25 μL) was first mixed with the DCM solution of BLG-NCA (6.6 mg, 0.025 mmol), into which the DCM solution of Hex-NH_2 (0.01 M, 25 μL) was added to start polymerization ($[M]_0 = 50$ mM, $[I]_0 = [CE]_0 = 0.5$ mM).

After the full conversion of monomers as confirmed by FTIR, the resulting polymers were purified by precipitation in hexane/ether (1:1, v/v) and dried under vacuum.

To monitor the polymerization kinetics, the polymerization solution was transferred into liquid FTIR cell after mixing. FTIR spectrum was collected at different time intervals until the disappearance of anhydride peaks from NCAs at 1865 and 1793 cm^{-1}. The conversion of NCA was calculated based on the standard curve of anhydride peak at 1793 cm^{-1} (Supplementary Fig. 17). Additionally, the polymerization kinetics was also monitored by NMR. The polymerization mixture in CD_2Cl_2 was transferred into an NMR tube, where the ^{1}H NMR spectrum was collected at different time intervals. The conversion of NCA was quantified by monitoring the integral of α-H of NCA (4.57-4.29 ppm, depending on the conversion). For the early-stage secondary structure study of CE-catalyzed polymerization, the polymerization mixture was diluted 10 times in DCM at different time intervals, and the resulting solution was measured by CD ([I] = 0.05 mM in the CD cuvette). Due to the absorbance of DCM, only the CD spectra at $\lambda > 220$ nm were measured.

To test the livingness of the CE-catalyzed polymerization, the conversion of NCA at different time points was first quantified by FTIR standard curve (1793 cm^{-1}). Several parallel polymerizations were prepared, which were stopped at different time through the addition of quench solution. The quench solution was prepared by mixing acetonitrile (ACN) and distilled (DI) water (1:1, v/v), followed by the addition of 1 vol% of HCl (37 wt%)[5]. The polypeptides in the quenched mixture were purified by precipitation in hexane/ether (1:1, v/v) and characterized by GPC.

CE-catalyzed polymerization of non-purified NCAs in a biphasic mixture

Non-purified BLG-NCA was prepared according to literature procedures[6]. The obtained non-purified NCA was dissolved in DCM and mixed with aqueous buffer (pH = 9.0) for 10 s, into which the DCM solution of Hex-NH_2 and 18-C-6 was added.

Synthesis of α,γ-dibenzyl-L-glutamate (DBLG)

The small-molecular analogue of propagating PBLG, α,γ-dibenzyl-L-glutamate (DBLG), was prepared by neutralizing the salt form of DBLG. Briefly, α,γ-dibenzyl-L-glutamate 4-toluenesulfonate (1.00 g, 2.00 mmol) was added into DCM or $CDCl_3$ (21 mL) at 0 °C and stirred for 30 min. NaOH (1 M, 21 mL) was added dropwise and stirred for another 2 h. The organic phase was separated, washed twice with DI water (21 mL), and dried over anhydrous Na_2SO_4. The obtained DBLG was stored in DCM or $CDCl_3$ solution at -20 °C to avoid the amidation of benzyl esters (Yield ~ 92%).

^{1}H NMR ($CDCl_3$, δ, 500 MHz): 7.41-7.29 (m, 10 H), 5.15 (s, 2H), 5.11 (s, 2H), 3.51 (dd, J = 8.4 and 5.1 Hz, 1 H), 2.51 (t, J = 7.6 Hz, 2H), 2.13-1.88 (m, 2H),1.51 (br s, 2H). ^{13}C NMR ($CDCl_3$, δ, 125 MHz): 175.6, 173.1, 136.1, 135.7, 128.8, 128.7, 128.6, 128.4, 128.4, 128.4, 67.0, 66.5, 54.0, 30.8, 29.8. HRMS (ESI, m/z): $[M+H]^+$ Calcd. for $C_{19}H_{22}NO_4$ 328.1549; Found: 328.1534.

Determination of activation energy

The reaction kinetics of CE-catalyzed ring-opening reaction of BLG-NCA in $CDCl_3$ was monitored at 293.15, 303.15, 313.15, and 323.15 K. $CDCl_3$ was selected as the solvent for the variable temperature (VT) NMR studies due to its higher boiling point. The $CDCl_3$ solution of DBLG (or the mixture of DBLG and 18-C-6) was first equilibrated for 10 min at target temperature, followed by the addition of $CDCl_3$ solution of BLG-NCA (final concentration was 30 mM, 3 mM and 3 mM for DBLG, BLG-NCA, and 18-C-6, respectively). The consumption of BLG-NCA was quantified by the integral of α-H in BLG-NCA (4.53-4.15 ppm, depending on the conversion and temperature), which was then used to calculate the apparent rate constant, k_{app}. The activation energy was determined from the $-\ln(k_{app})$ *vs* 1/T Arrhenius plot.

DOSY studies

The DOSY spectra of BLG-NCA, 18-C-6, $PBLG-NH_2$, PBLG-NHAc, or their mixtures were

collected. While the bimolecular DOSY spectra were collected at room temperature (25 °C), the termolecular DOSY spectra were collected at -14 °C to minimize the CE-catalyzed ring-opening reaction of BLG-NCA. For the termolecular DOSY study, the $CDCl_3$ solution of BLG-NCA was added into the inner chamber of the reaction monitoring system NMR tube (Wilmad-LabGlass, Vineland, NJ, USA), and the mixture of $CDCl_3$ solution of PBLG-NH_2 and 18-C-6 was added into the outer chamber. The inner and outer chamber was separated by a Teflon tip to ensure no mixing. Once the temperature was equilibrated to -14 °C the DOSY spectrum before mixing was collected. The inner chamber tube was then removed to allow for the mixing of two solutions. The DOSY spectrum after mixing was then collected. The ^{1}H NMR spectra before and after mixing confirmed the negligible ring-opening reaction of BLG-NCA, by quantifying the integral of α-proton of BLG-NCA at ~ 4.40 ppm.

DOSY experiments were performed on an Agilent VNMRS 750MHz spectrometer with a 5mm indirect-detection triple resonance probe with Z-gradient (PFG) capability. The DOSY spectra were collected with gradient compensated stimulated echo and convection compensated pulse sequence (DgsteSL_cc) with a trim pulse of 2 ms, a diffusion gradient duration of 2 ms, a diffusion delay of 50 ms, and 15 gradient field strengths in the range of 2.70 to 35.31 G/cm or 2.70-39.46 G/cm. The data were processed with Agilent VNMRJ4.2A software. A line broadening of 1 Hz, phase and baseline correction were performed before data processing. The diffusion coefficients were extracted by fitting the integral values of the arrayed spectra to the Stejskal-Tanner function[7, 8] (Supplementary Eq. 1):

$$I = I_0 \exp\left[-D\gamma^2 G^2 \delta^2 \left(\Delta - \frac{\delta}{3}\right)\right] \quad (1)$$

where I is the intensity (Integral) of a peak; G is the gradient strength, δ is the gradient duration, and Δ is the diffusion delay.

Determination of binding stoichiometry

The binding stoichiometry of 18-C-6 and BLG-NCA was determined by continuous variation method[9-11]. The CD_2Cl_2 solution of 18-C-6 and BLG-NCA were mixed at different ratios with

a constant total concentration (*i.e.*, 10 mM). The NMR spectra of all samples were collected and analyzed by Job plot method[12]. The binding stoichiometry was determined with the largest change in [NCA]*$\Delta\delta$ plotted against the molar fraction of NCA, where $\Delta\delta$ *is* the change in chemical shift of ring N-H proton of BLG-NCA (~6.03 ppm).

Kinetics analysis of ring-opening reaction

The ring-opening reaction of BLG-NCA by DBLG was investigated in the absence and presence of 18-C-6, which mimicked the ring-opening reaction of BLG-NCA during the chain propagation step. The $CDCl_3$ solution of the DBLG, 18-C-6, and BLG-NCA were mixed, and the conversion of BLG-NCA over time was monitored by ^{1}H NMR. The conversion of NCA was quantified by monitoring the integral of α-H of NCA. The real concentrations of DBLG and 18-C-6 was normalized according to the integral of corresponding peaks.

For the ring-opening reaction in the absence of 18-C-6, the reaction order in DBLG was determined by monitoring the kinetics at different $[DBLG]_0$ while fixing $[BLG\text{-}NCA]_0$ (*i.e.*, $[DBLG]_0/[BLG\text{-}NCA]_0$ = 5, 8, 10, 12, and 15). Since DBLG was in large excess, we assumed [DBLG] was constant during the test, which rendered the ring-opening reaction a pseudo first-order reaction for BLG-NCA (with an apparent rate constant, $k_{app} = k[DBLG]_0^m$, Supplementary Eq. 2). The plot of ln([NCA]) *vs* time showed a good linear relationship (Supplementary Fig. 18), validating our assumption of the pseudo first-order kinetics. The reaction order in DBLG (*m* in Supplementary Eq. 2) was calculated through the logarithmic plot of k_{app}-$[DBLG]_0$, which was determined to be (0.64 ± 0.05).

The reaction orders in DBLG and 18-C-6 in CE-catalyzed ring-opening reaction were determined in a similar way, but with $k_{app} = k[DBLG]_0^a[18\text{-}C\text{-}6]_0^b$ (Supplementary Eq. 3). For the determination of reaction order in DBLG, the NMR kinetics were collected at different $[DBLG]_0$ while fixing $[BLG\text{-}NCA]_0$ and $[18\text{-}C\text{-}6]_0$ (*i.e.*, $[DBLG]_0/[BLG\text{-}NCA]_0$ = 5, 8, 10, 12, and 15); for the determination of reaction order in 18-C-6, the NMR kinetics were collected at different $[18\text{-}C\text{-}6]_0$ while fixing $[BLG\text{-}NCA]_0$ and $[DBLG]_0$ (*i.e.*, $[18\text{-}C\text{-}6]_0/[BLG\text{-}NCA]_0$ = 1/3, 2/3, 1, 2, and 3).

$$-\frac{d[NCA]}{dt} = k[DBLG]_0^m [NCA] = k_{app}[NCA] \qquad (2)$$

$$-\frac{d[NCA]}{dt} = k[DBLG]_0^a [18\text{-C-6}]_0^b [NCA] = k_{app}[NCA] \qquad (3)$$

The reaction order in DBLG (*a* in Supplementary Eq. 3) and 18-C-6 (*b* in Supplementary Eq. 3) was determined to be (0.50 ± 0.06) and (0.26 ± 0.03), respectively.

All experimental conditions for the determination of reaction orders were summarized in Supplementary Table 3.

Molecular dynamics simulation

All-atom structures, topologies, and interaction potentials for 18-C-6, DBLG, BLG-NCA, and DCM solvent were constructed using the Automated Topology Builder (ATB) server (http://atb.uq.edu.au)[13]. Initial molecular geometries were constructed using density functional theory at the B3LYP/6-31G* level of theory[14] in implicit PCM solvent[15]. Initial partial charges assigned using the Kollman-Singh method[16] and further optimized within ATB by enforcing symmetries and defining charge groups[13]. All molecules carried zero net charge. Bonded and non-bonded parameters were specified according to the Groningen Molecular Simulation (GROMOS) 54A7 force field[17].

All-atom molecular dynamics simulations were conducted using the Gromacs 2019 simulation suite[18]. Lennard-Jones interactions were smoothly shifted to zero at 1.4 nm and unlike atom interactions dictated by Lorentz-Berthelot combining rules[19]. Electrostatics were treated by Particle Mesh Ewald (PME) with a real-space cutoff of 1.4 nm and a 0.12 nm grid spacing in reciprocal space[20]. Tail corrections in energy and pressure were applied. Bond lengths were fixed to their equilibrium lengths using the LINCS algorithm[21]. Net center of mass motion was removed every 10 ps. Simulations were conducted in the NPT ensemble at 298 K and 1 bar. Temperature control was maintained using a Nosé-Hoover thermostat[22] with a 0.5 ps time constant, and pressure control maintained using an isotropic Parrinello-Rahman barostat[23] with a 4 ps time constant and a compressibility of 4.5×10^{-5} bar^{-1}. Periodic boundary conditions were employed in all three dimensions. Newton's equations of motion were integrated using the

leap-frog algorithm with a 2 fs time step[24]. Simulation trajectories were visualized using Visual Molecular Dynamics[25].

Two simulations were conducted. In the first, an 18×18×18 nm^3 box was constructed into which 105 DBLG and 11 BLG-NCA molecules were inserted and bathed in 51,920 DCM solvent molecules. This corresponds to a DBLG concentration of 30 mM and a BLG-NCA concentration of 3 mM. In the second, 105 DBLG, 11 BLG-NCA, and 11 18-C-6 molecules were inserted and bathed in 51,839 DCM solvent molecules. This corresponds to a DBLG concentration of 30 mM, a BLG-NCA concentration of 3 mM, and an 18-C-6 concentration of 3 mM. The second system differs from the first only in the addition of 18-C-6 molecules in a 1:1 ratio with BLG-NCA and a commensurate reduction in the number of DCM solvent molecules to account for the additional volume of CE.

Each system was then subjected to the same simulation and analysis protocol. The system was first subjected to the steepest descent energy minimization until the maximum force on any given atom fell below a threshold of 100 kJ/mol·nm. Initial atomic velocities were then assigned from a Maxwell-Boltzmann distribution at 298 K and a 100 ps NPT equilibration run at 298 K and 1 bar was conducted to relax and stabilize the system. Finally, a 10 ns NPT production run at 298 K and 1 bar was performed and system configurations saved every 10 ps for analysis. The production runs were analyzed using in-house Python tools employing the MDAnalysis libraries[26, 27] that computed the center of geometry and atomic pairwise distances between the 11 DBLG molecules and the 11 BLG-NCA molecules. Radial distribution functions were computed in the center of geometry distances $g(r_{\text{DBLG-BLG-NCA}})$ were calculated using a Δr = 0.075 nm bin spacing. Uncertainties were estimated by partitioning the production run into five equal sized blocks and computing standard deviations over the blocks. Pairwise distances between the atoms constituting DBLG and BLG-NCA were computed for pairs of molecules with a center-of-geometry distance $r_{\text{DBLG-BLG-NCA}} < 1$ nm, and contact matrices for these contact pairs computed by averaging over all 5774 such configurations observed in the saved snapshots of the production run without 18-C-6 and 7129 configurations in that with 18-C-6. Again, uncertainties were estimated by partitioning the production run into five equal sized blocks and computing standard deviations over the blocks. The radial distribution

functions reveal differences in the molecular association probabilities of DBLG and BLG-NCA induced by the 18-C-6, and the configuration-averaged contact matrices reveal differences in the molecular geometry of approach of DBLG and BLG-NCA within the DBLG/BLG-NCA contact pairs.

Supplementary Figures

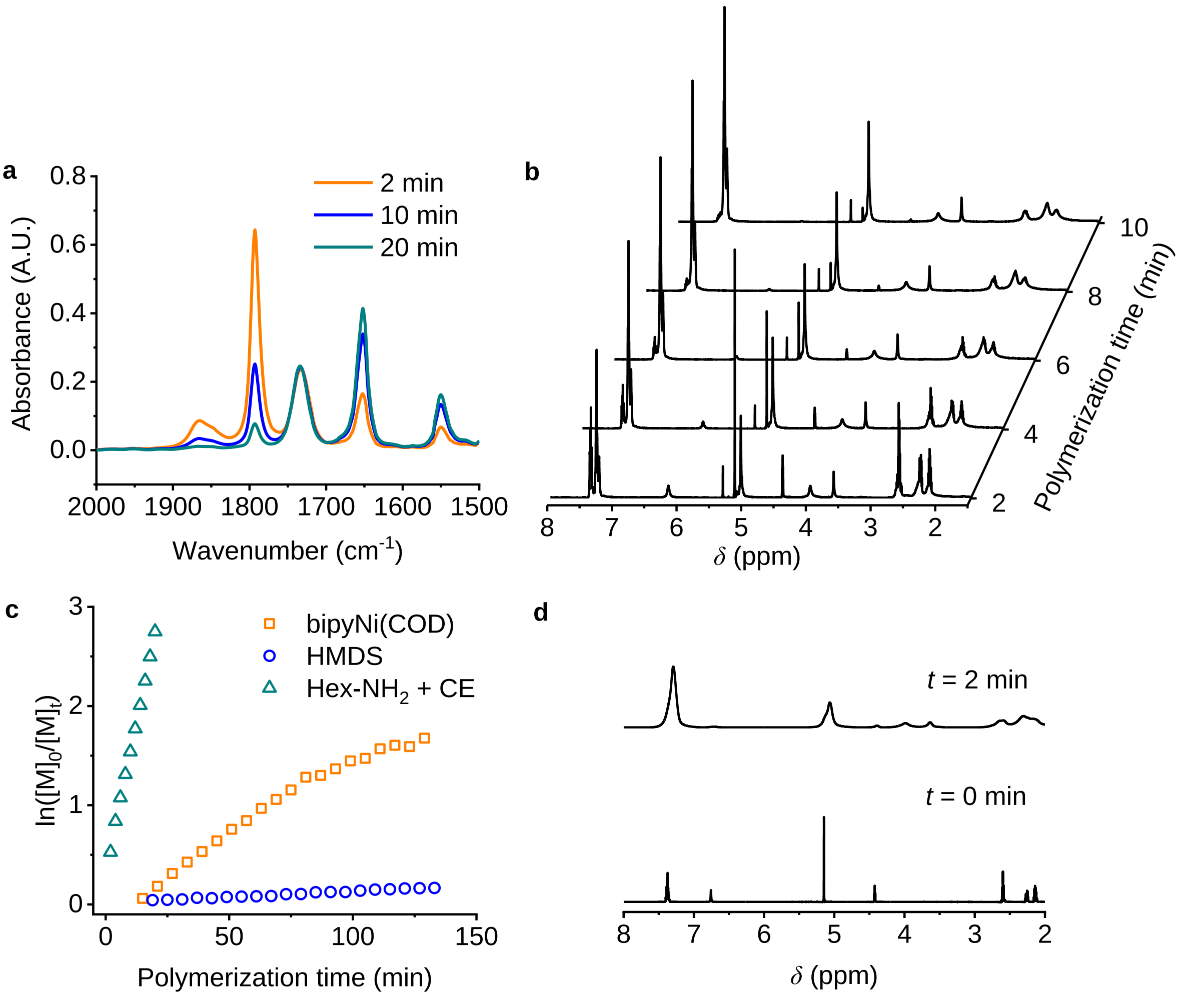

Supplementary Figure 1. Kinetic analysis of CE-catalyzed polymerization of NCA. **a** Overlaid FTIR spectra showing the CE-catalyzed polymerization of BLG-NCA in DCM initiated by Hex-NH_2. $[M]_0/[I]_0 = 100$, $[I]_0 = [CE]_0 = 0.5$ mM. **b** Overlaid ^1H NMR spectra showing the CE-catalyzed polymerization of BLG-NCA in CD_2Cl_2 initiated by Hex-NH_2. $[M]_0/[I]_0 = 100$, $[I]_0 = [CE]_0 = 0.5$ mM. **c** Semilogarithmic kinetic plot of polymerization of BLG-NCA initiated by bipyNi(COD) in THF and by HMDS in DMF. $[M]_0/[I]_0 = 100$, $[M]_0 = 200$ mM. The CE-catalyzed kinetics at a lower $[M]_0$ (*i.e.*, 50 mM) in DCM was provided for comparison. **d** Overlaid ^1H NMR spectra showing the CE-catalyzed polymerization of BLG-NCA in CD_2Cl_2 initiated by Hex-NH_2 at high $[M]_0$. $[M]_0/[I]_0 = 100$, $[I]_0 = [CE]_0 = 4$ mM.

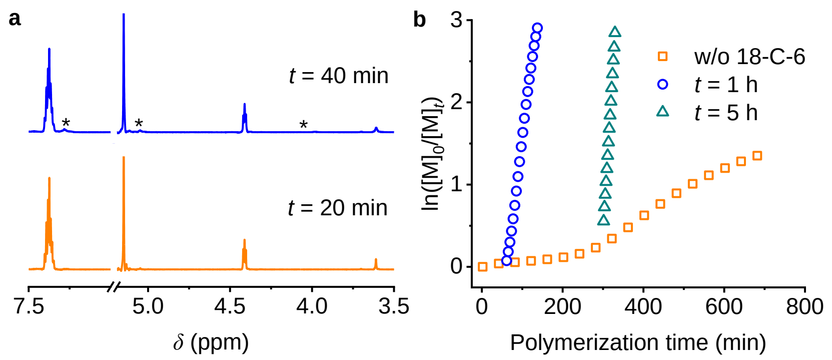

Supplementary Figure 2. The role of CE in the fast polymerization of NCA. **a** Overlaid NMR spectra showing the stability of BLG-NCA in CD_2Cl_2 in the presence of 18-C-6. The stars indicate the peaks from degradation product (PBLG oligomers). [NCA] = 50 mM, [CE] = 0.5 mM. **b** Semilogarithmic kinetic plot of polymerization of BLG-NCA in DCM initiated by Hex-NH_2 with the addition of 18-C-6 at different time points. CE was added during the first stage (*i.e.*, propagating PBLG as random coils with slow polymerization rate, t = 1 h) as well as the second stage (*i.e.*, propagating PBLG as α-helices with fast polymerization rate, t = 5 h). The Hex-NH_2-initiated polymerization in DCM in the absence of 18-C-6 was plotted for comparison. $[M]_0/[I]_0 = 100$, $[I]_0 = [CE]_0 = 0.5$ mM.

BLG-NCA is stable for at least 40 min (8% degradation), ruling out the possibility that 18-C-6 served as an initiator. The degradation at 40 min likely resulted from trace amount of water that opened the NCA ring, which released an amino group that induced oligomerization of BLG-NCA under the catalysis of 18-C-6. Additionally, the catalytic effect of CE was independent on the type of amino groups (*i.e.*, Hex-NH_2 when CE was mixed before the initiation, random-coiled propagating chains when CE was added at t = 1 h, or α-helical propagating chains when CE was added at t = 1 h). This result excludes the possibility that 18-C-6 served as a co-initiator.

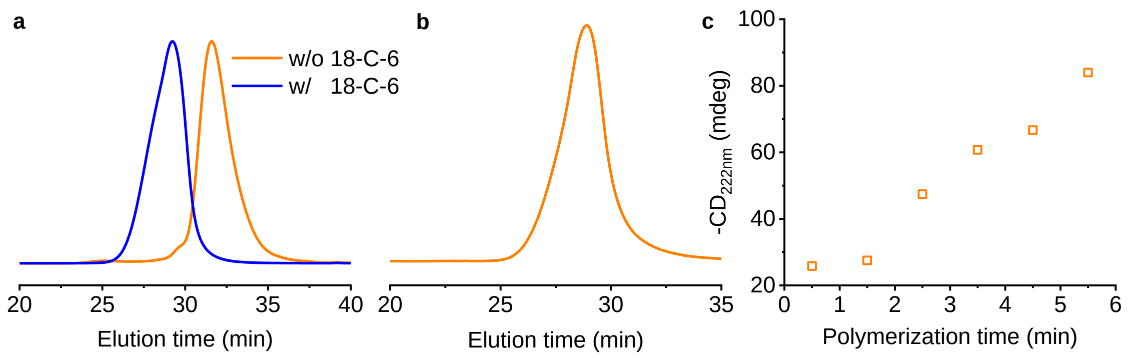

Supplementary Figure 3. Cooperative polymerization of NCA catalyzed by CE. **a** Normalized GPC-LS traces of the obtained PBLG initiated by Hex-NH_2 in the presence and absence of 18-C-6. **b** GPC-dRI trace of the obtained PBLG initiated by Hex-NH_2 in the presence of 18-C-6. **c** The change in ellipticity at 222 nm over time for CE-catalyzed polymerization of BLG-NCA initiated by Hex-NH_2 in DCM. $[M]_0/[I]_0 = 100$, $[I]_0 = [CE]_0 = 0.5$ mM.

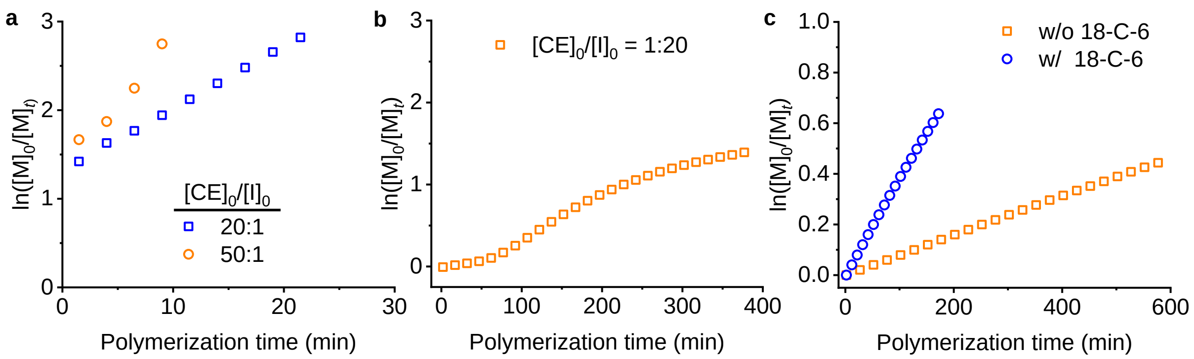

Supplementary Figure 4. The impact of $[CE]_0/[I]_0$ ratios and monomer chirality on the rate acceleration. **a**, **b** Semilogarithmic kinetic plot of polymerization of BLG-NCA in DCM initiated by Hex-NH_2 with different $[CE]_0/[I]_0$ ratios. $[M]_0/[I]_0 = 100$, $[I]_0 = 0.5$ mM. **c** Semilogarithmic kinetic plot of polymerization of racemic BDLG-NCA in DCM initiated by Hex-NH_2 in the presence and absence of 18-C-6. $[M]_0/[I]_0 = 100$, $[I]_0 = [CE]_0 = 0.5$ mM.

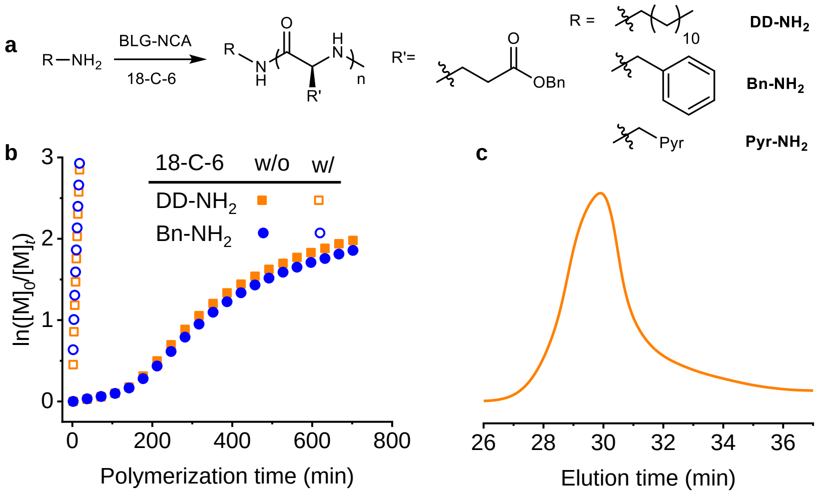

Supplementary Figure 5. Preparation of *C*-terminus-functionalized polypeptides with CE-catalyzed polymerization of NCA. **a** Scheme illustrating CE-catalyzed polymerization of NCA from various small molecular amine initiators. **b** Semilogarithmic kinetic plot of polymerization of BLG-NCA in DCM initiated by various small molecular initiators in the presence and absence of 18-C-6. **c** GPC-UV trace (λ = 345 nm) of the obtained PBLG initiated with Pyr-NH_2 in the presence of 18-C-6. The monomodal peak suggested the successful functionalization of *C*-terminus. $[M]_0/[I]_0 = 100$, $[I]_0 = [CE]_0 = 0.5$ mM.

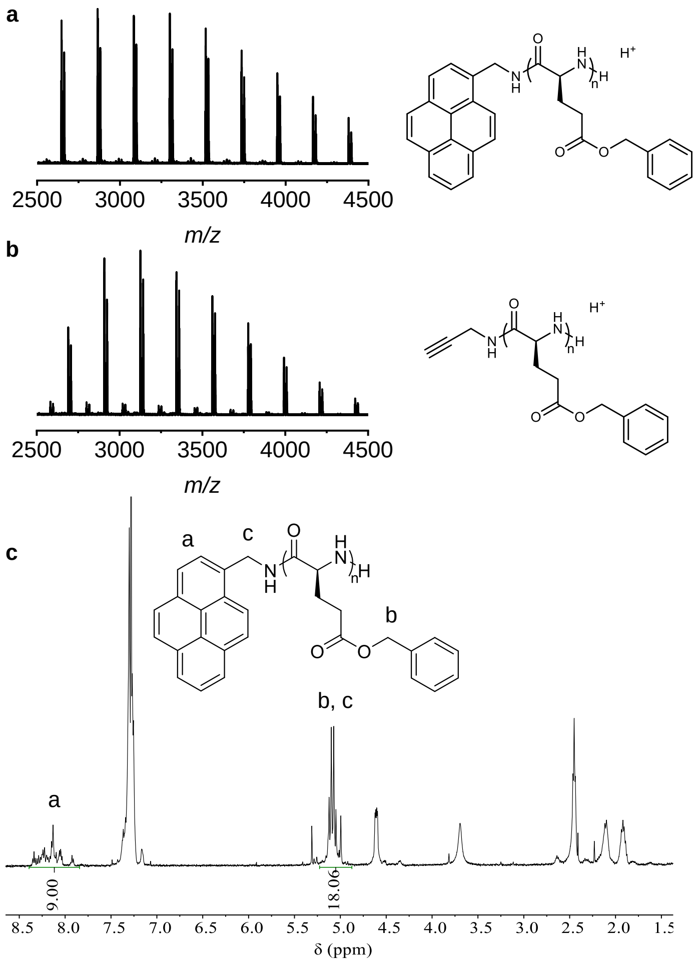

Supplementary Figure 6. Confirmation of functionalization of *C*-terminus from CE-catalyzed polymerization. **a**, **b** MALDI-TOF MS characterization of obtained PBLG initiated by Pyr-NH_2 (**a**) and PP-NH_2 (**b**) in the presence of 18-C-6. The obtained *m*/*z* signals agree well with the calculated values, which are (232.11 + 219.09n) and (249.14 + 219.09n) ($[M+H]^+$ and $[M+NH_4]^+$) for Pyr-NH_2-initiated polypeptides and (56.05 + 219.09n) and (73.08 + 219.09n) ($[M+H]^+$ and $[M+NH_4]^+$) for PP-NH_2-initiated polypeptides. The chemical structures corresponding to the $[M+H]^+$ species were provided next to the MALDI-TOF MS spectrum. **c** ^1H NMR spectrum of obtained PBLG initiated by Pyr-NH_2 in $CDCl_3$/TFA-*d* (85:15, v/v). The peaks at 8.39-7.84 ppm corresponds to the protons from Pyr-NH_2 initiator. $[M]_0/[I]_0 = 25$, $[I]_0 = [CE]_0 = 1$ mM. The polymerization was stopped at 40 s for easier characterization.

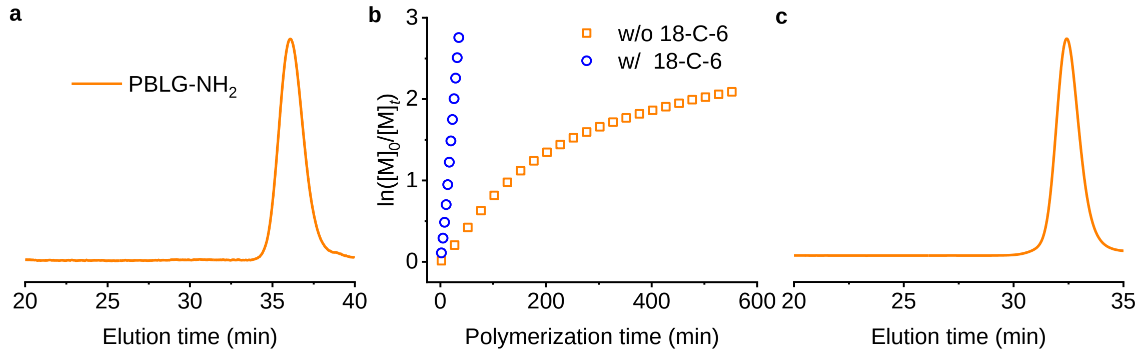

Supplementary Figure 7. CE-catalyzed polymerization of BLG-NCA initiated by α-helical PBLG macroinitiator. **a** GPC-LS trace of α-helical PBLG macroinitiator. M_n = 7.2 kDa, $Đ$ = 1.05. **b** Semilogarithmic kinetic plot of polymerization of BLG-NCA in DCM initiated by α-helical PBLG macroinitiator in the presence and absence of 18-C-6. **c** GPC-dRI trace of the obtained PBLG initiated by α-helical PBLG macroinitiator in the presence of 18-C-6. $[M]_0/[I]_0$ = 100, $[I]_0$ = $[CE]_0$ = 1 mM.

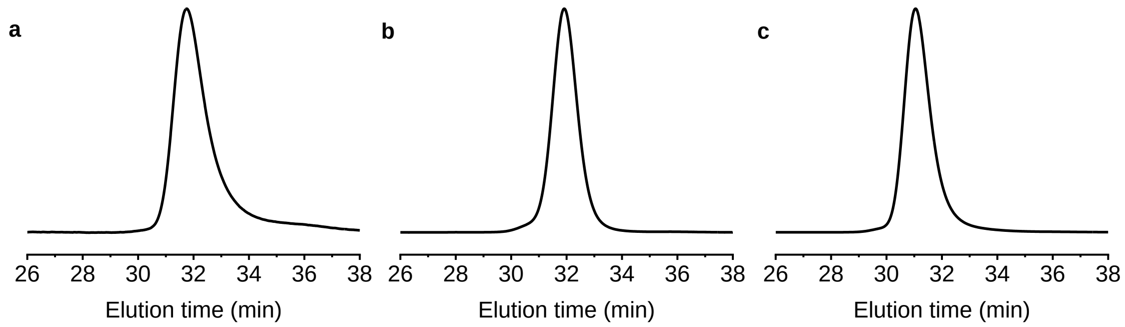

Supplementary Figure 8. GPC-LS traces of the obtained polypeptides initiated by α-helical PBLG macroinitiator in the presence 18-C-6. **a** ELG-NCA, **b** ZLL-NCA, **c** POB-NCA. $[M]_0/[I]_0 = 100$, $[I]_0 = [CE]_0 = 1$ mM.

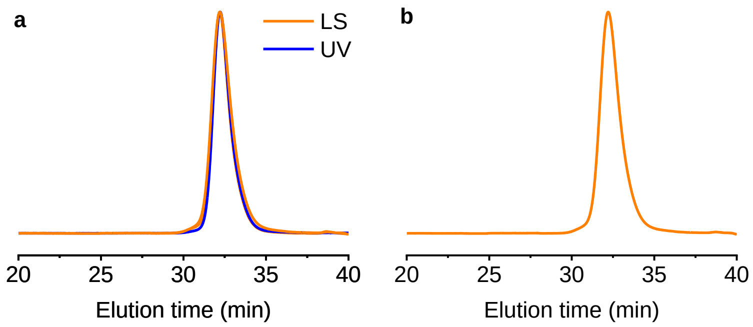

Supplementary Figure 9. Preparation of *C*-terminus-functionalized polypeptides with predictable MWs. (**a**) GPC-LS and GPC-UV ($\lambda = 345$ nm) traces of the obtained PBLG initiated by Pyr-functionalized α-helical PBLG macroinitiators in the presence 18-C-6. M_n = 36.2 kDa, $Đ$ =1.05. (**b**) GPC-LS trace of the obtained PBLG initiated by PP-functionalized α-helical PBLG macroinitiators in the presence 18-C-6. M_n = 33.9 kDa, $Đ$ = 1.05. $[M]_0/[I]_0 = 100$, $[I]_0 = [CE]_0 = 1$ mM.

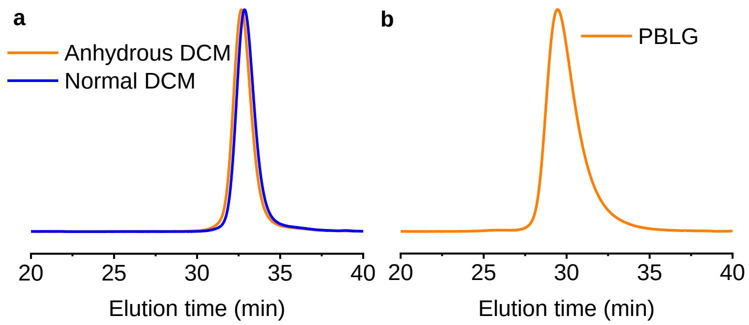

Supplementary Figure 10. Minimal side reactions with CE-catalyzed polymerization of NCA. **a** Normalized GPC-LS traces of the obtained polypeptides initiated by α-helical PBLG macroinitiator in the presence 18-C-6 in anhydrous DCM and normal DCM. $[M]_0/[I]_0 = 100$, $[I]_0 = [CE]_0 = 1$ mM. **b** GPC-LS trace of obtained polypeptides from the CE-catalyzed polymerization of non-purified BLG-NCA initiated by Hex-NH_2 in a biphasic reaction. $[M]_0/[I]_0 = 100$, $[I]_0 = [CE]_0 = 0.5$ mM, DCM:water = 10:1 (w/w). $M_n = 74.8$ kDa, $Đ = 1.19$.

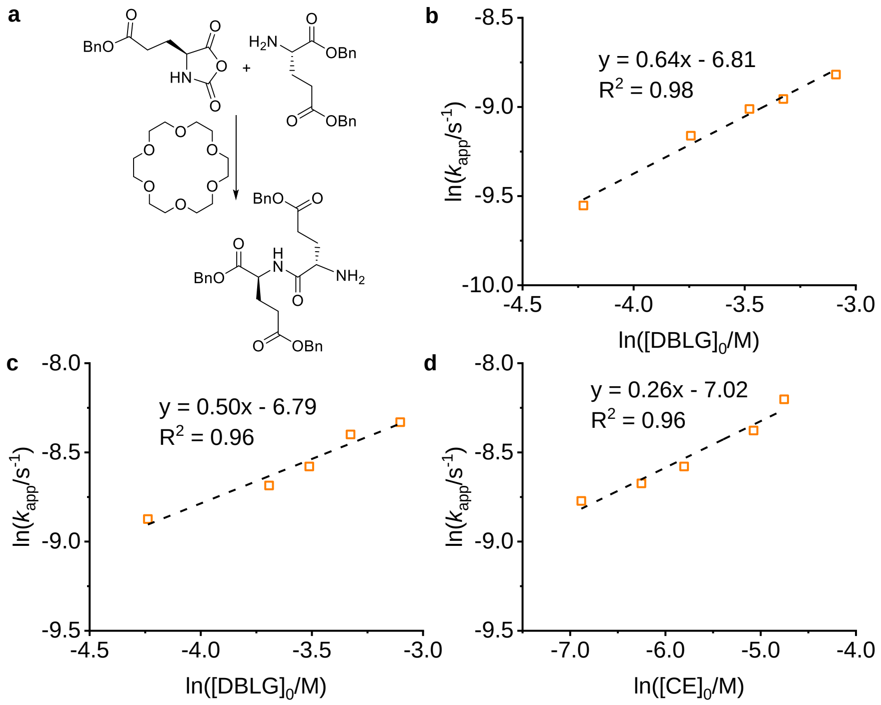

Supplementary Figure 11. Determination of reaction orders for ring-opening reaction of BLG-NCA by DBLG. **a** Scheme illustrating CE-catalyzed ring-opening reaction of BLG-NCA by DBLG, which mimics the ring-opening reaction during chain propagation step. **b-d** Determination of reaction order with respect to DBLG in the absence of 18-C-6 (**b**), with respect to DBLG in the presence of 18-C-6 (**c**), and with respect to 18-C-6 (**d**).

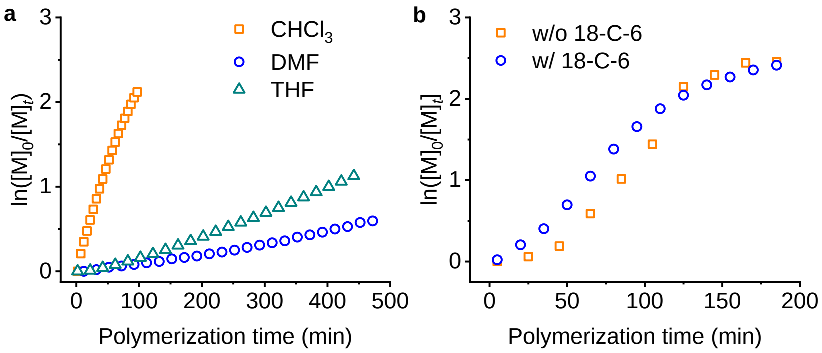

Supplementary Figure 12. Elucidation of mechanism of CE-catalyzed polymerization of NCA. **a** Semilogarithmic kinetic plot of polymerization of BLG-NCA in various solvents initiated by Hex-NH_2 in the presence of 18-C-6. **b** Semilogarithmic kinetic plot of polymerization of BLG-NCA in DCM initiated by HMDS in the presence and absence of 18-C-6. $[M]_0/[I]_0 = 100$, $[I]_0 = [CE]_0 = 0.5$ mM.

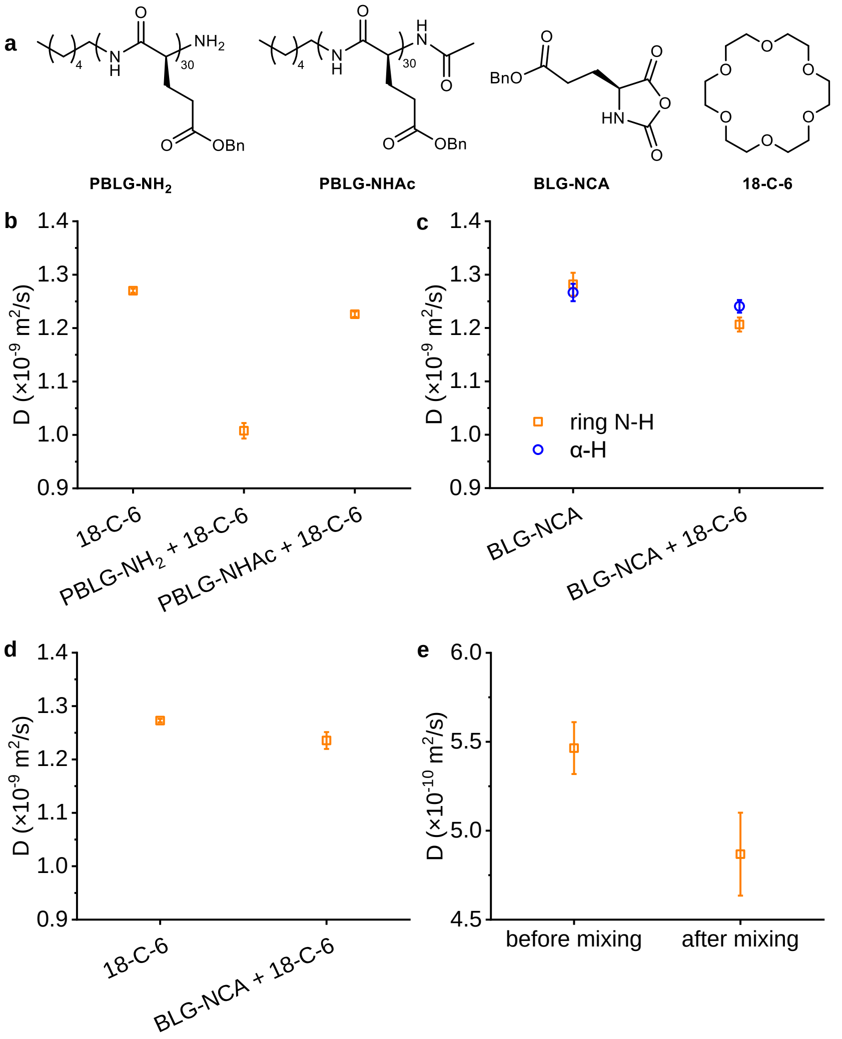

Supplementary Figure 13. Diffusion coefficients of 18-C-6 and BLG-NCA under various conditions as determined by DOSY. **a** Chemical structures of PBLG-NH_2, PBLG-NHAc, BLG-NCA, and 18-C-6 used in this study. **b** Diffusion coefficients of 18-C-6 (0.3 mM) in the presence of PBLG-NH_2 or PBLG-NHAc (0.3 mM) in CD_2Cl_2. **c** Diffusion coefficients of BLG-NCA (10 mM) in the presence of 18-C-6 (10 mM) in CD_2Cl_2. **d** Diffusion coefficients of 18-C-6 (10 mM) in the presence of BLG-NCA (10 mM) in CD_2Cl_2. **e** Diffusion coefficients of BLG-NCA (1 mM) before and after mixing with the solution of PBLG-NH_2 (1 mM) and 18-C-6 (1 mM) at -14 °C in $CDCl_3$. Error bars represent the errors generated during the fitting of the arrayed spectra with the Stejskal-Tanner function using VnmrJ.

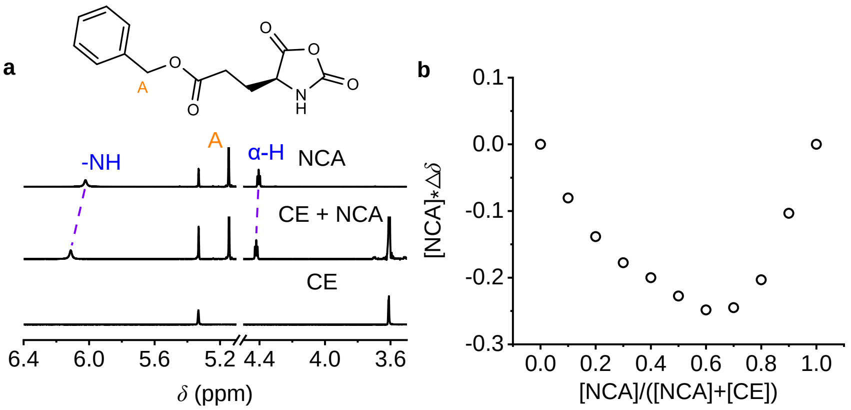

Supplementary Figure 14. NMR titration of BLG-NCA with 18-C-6. **a** Representative NMR spectra illustrating the chemical shift of peaks from BLG-NCA (10 mM) with the addition of 18-C-6 (5 mM). **b** Job plot for the determination of binding stoichiometry between BLG-NCA and 18-C-6. [BLG-NCA] + [18-C-6] = 10 mM, $T = 298$ K.

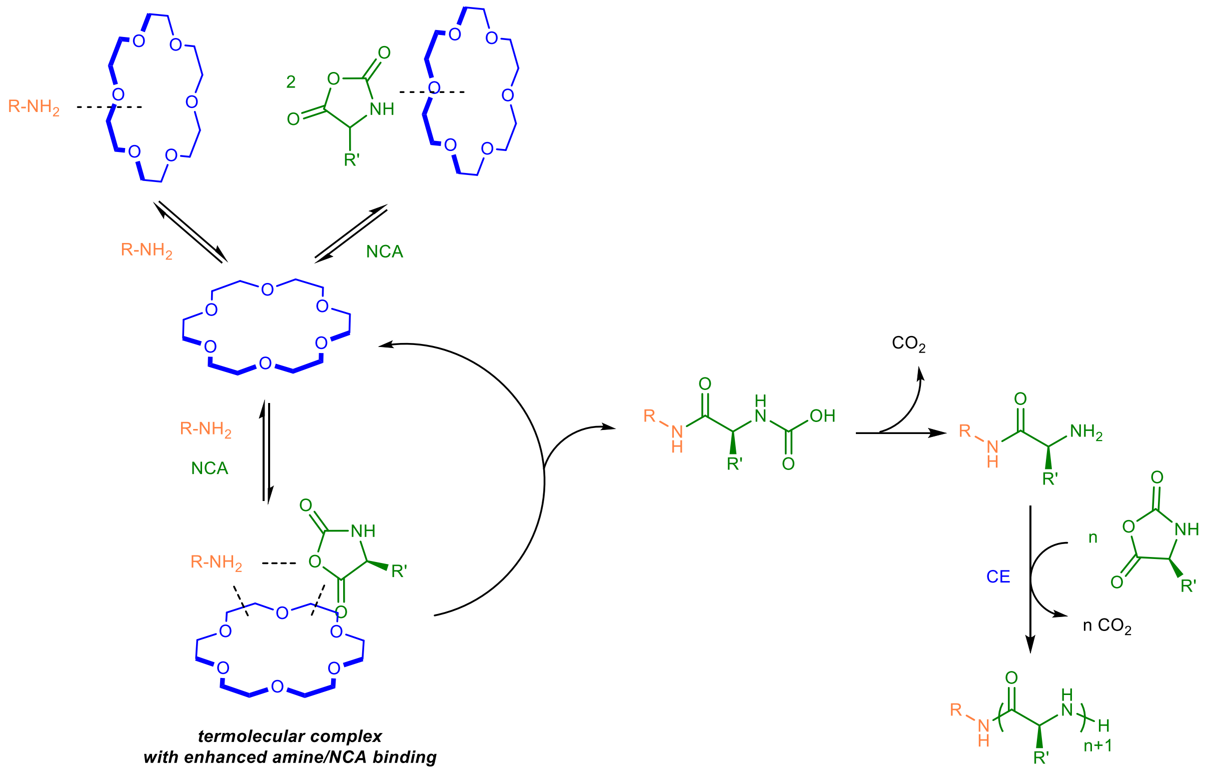

Supplementary Figure 15. Proposed mechanism illustrating the role of CE (18-C-6 as an example) in the accelerated polymerization of NCA.

a b

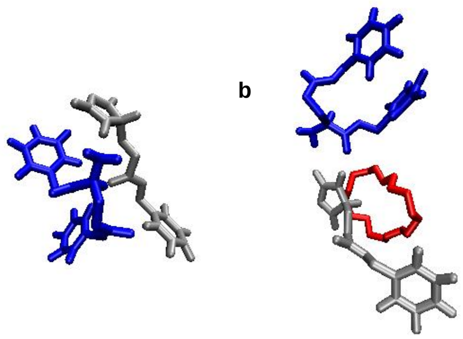

Supplementary Figure 16. Additional illustrative snapshots of a BLG-NCA and DBLG contact pair in a bimolecular complex (**a**) and in a termolecular complex comprising a mediating 18-C-6 molecule (**b**). The snapshots are colored under a molecular coloring scheme. DBLG = blue, BLG-NCA = silver, 18-C-6 = red.

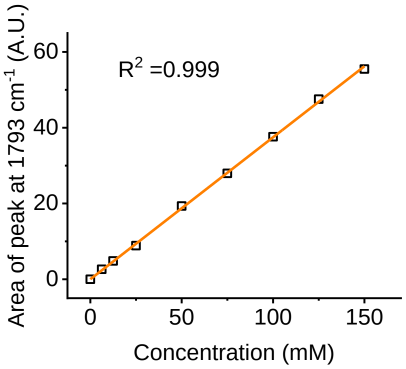

Supplementary Figure 17. Standard curve of the anhydride peak (1793 cm^{-1}) from BLG-NCA characterized by FTIR. A(1793 cm^{-1}, A.U.) = 0.375 c(BLG-NCA) (mM).

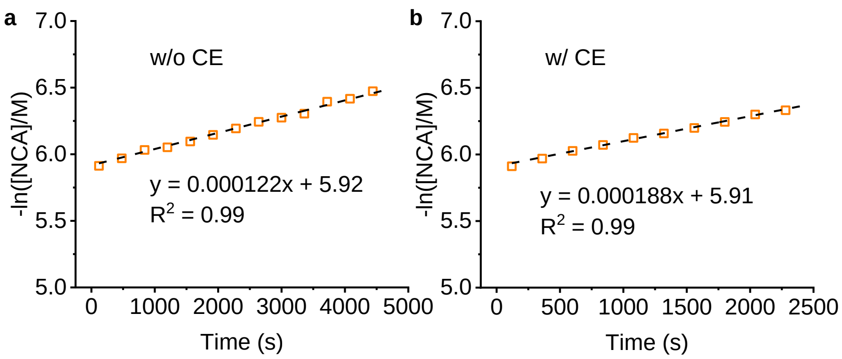

Supplementary Figure 18. Representative semilogarithmic kinetic plot of ring-opening reaction of BLG-NCA in $CDCl_3$ for the determination of reaction orders in the absence (**a**) and presence of CE (**b**). $[DBLG]_0$ = 30 mM, $[NCA]_0$ = $[CE]_0$ = 3 mM.

Supplementary Tables

Supplementary Table 1. Characterization of polypeptides from the convention polymerization in DMF or the CE-catalyzed polymerization in DCM.[a]

Entry	Solvent	$[I]_0$ (mM)	t (h)[b]	M_n/M_n* (kDa)[c,d]	$Đ$[d]
1	DCM	0.1	4.5	230/226	1.09
2	DMF	0.4	72	123/226	1.35

[a]The polymerizations initiated by α-helical PBLG macroinitiator were carried out in a glovebox at room temperature. For CE-catalyzed polymerization, 18-C-6 was used as catalyst. $[I]_0 = [CE]_0$, $[M]_0/[I]_0 = 1{,}000$. [b]Polymerization time reaching > 95% monomer conversion. [c]Obtained MWs/designed MWs*. [d]Determined by GPC; dn/dc = 0.100 and 0.098 for Entry 1 and 2, respectively.

Supplementary Table 2. Characterization of polypeptides from the CE-catalyzed polymerization in normal and anhydrous DCM.[a]

Entry	Solvent	M_n/M_n*(kDa)[b,c]	$Đ^c$
1	normal	28.7/29.2	1.05
2	anhydrous	30.7/29.2	1.05

[a]The polymerizations initiated by α-helical PBLG macroinitiator were carried out in the presence of 18-C-6 at room temperature. $[M]_0/[I]_0 = 100$, $[I]_0 = [CE]_0 = 1$ mM. [b]Obtained MWs/designed MWs*. [c]Determined by GPC; dn/dc = 0.098.

Supplementary Table 3. Reaction conditions and the calculated apparent rate constants (k_{app}) for the determination of reaction orders.[a]

Entry	$[DBLG]_0$ (mM)	$[CE]_0$ (mM)	$k_{app} \times 10^4$ (s^{-1})
1	15	3	1.40
2	24	3	1.69
3	30	3	1.88
4	36	3	2.25
5	45	3	2.41
6	30	1	1.55
7	30	2	1.71
8	30	6	2.30
9	30	9	2.74
10	15	-	0.71
11	24	-	1.05
12	30	-	1.22
13	36	-	1.29
14	45	-	1.48

[a]The ring-opening reaction was monitored in $CDCl_3$ at 25 °C by ^1H NMR. $[BLG\text{-}NCA]_0$ = 3 mM.

Supplementary Note

NMR spectra

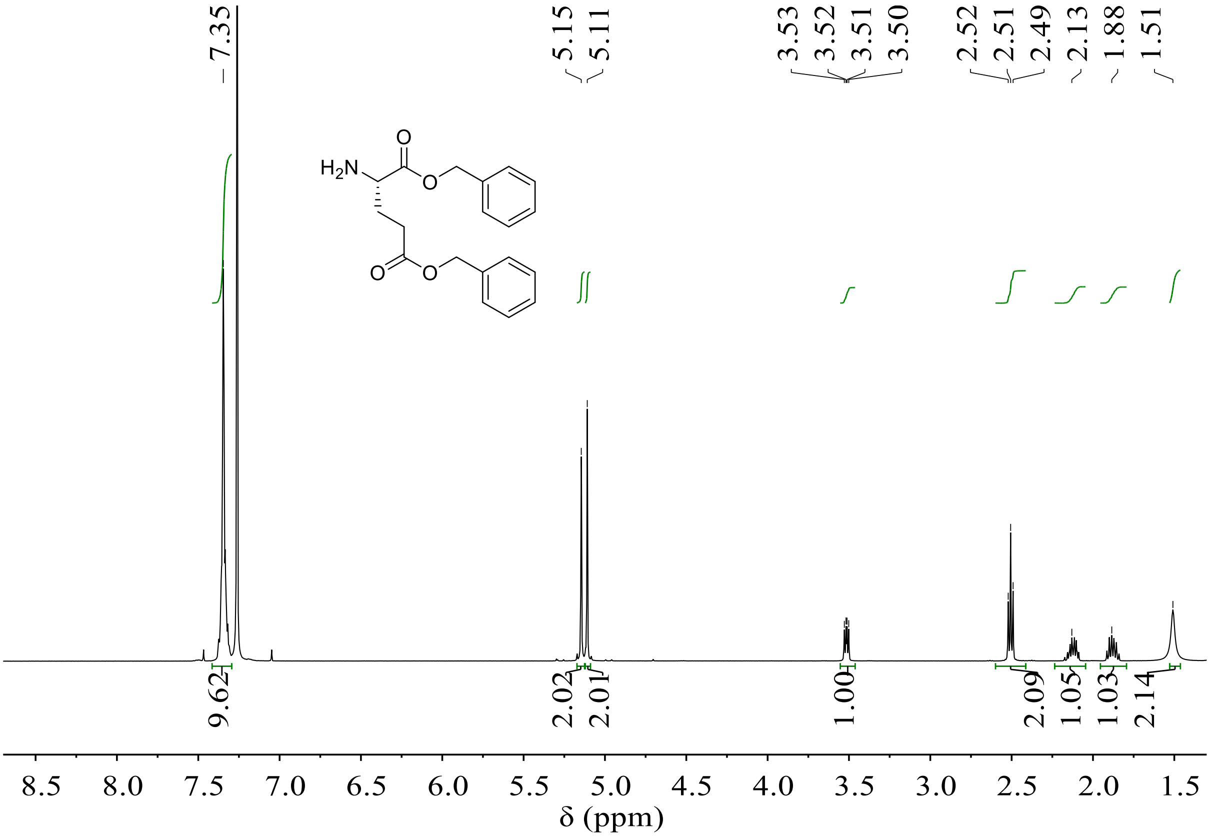

Supplementary Figure 19. ^{1}H NMR spectrum (500 MHz) of DBLG in $CDCl_3$.

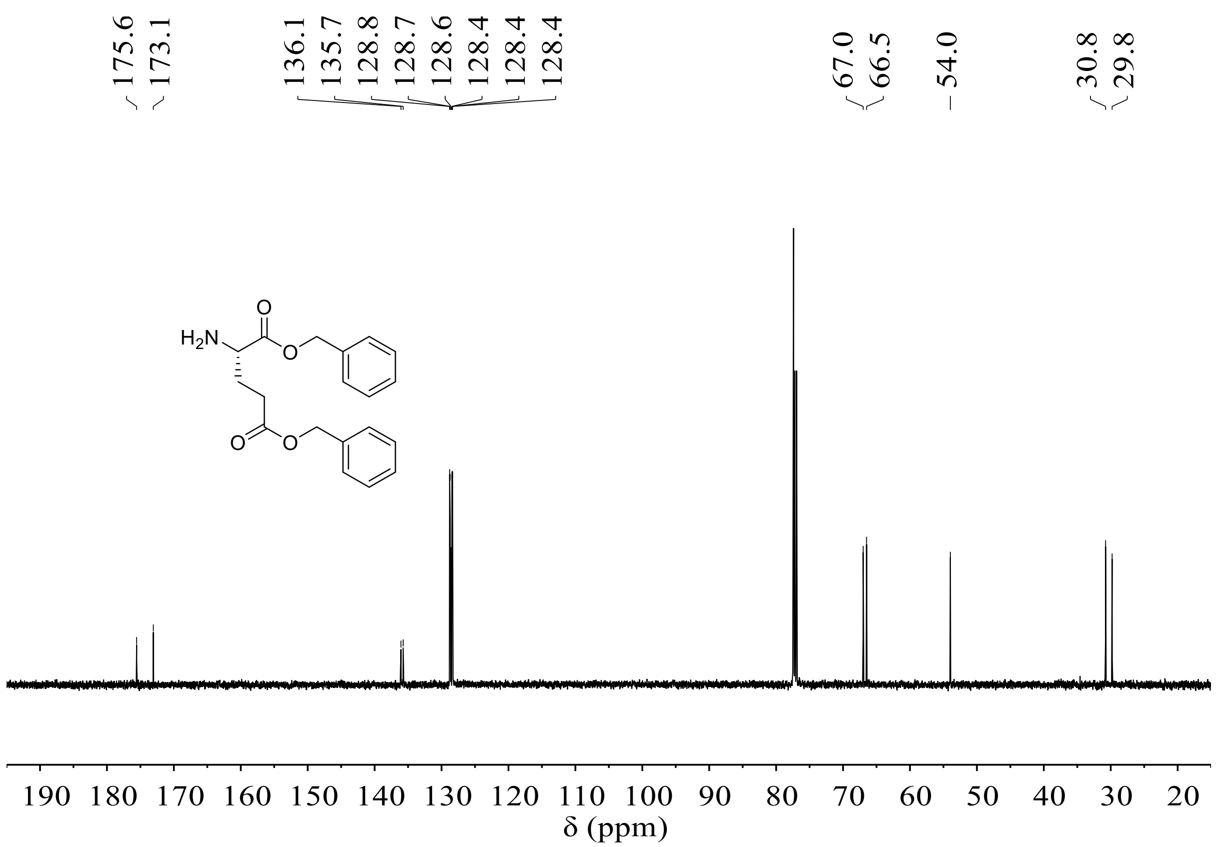

Supplementary Figure 20. ^{13}C NMR spectrum (125 MHz) of DBLG in $CDCl_3$.

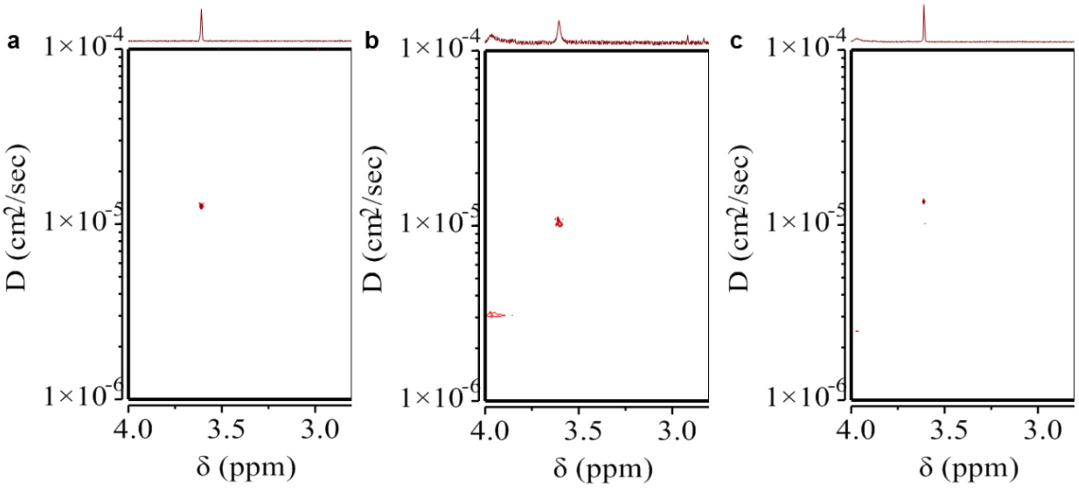

Supplementary Figure 21. DOSY spectra showing the diffusion coefficients of 18-C-6 in CD_2Cl_2. **a** in the absence of any polypeptides, **b** in the presence of PBLG-NH_2, and **c** in the presence of PBLG-NHAc.

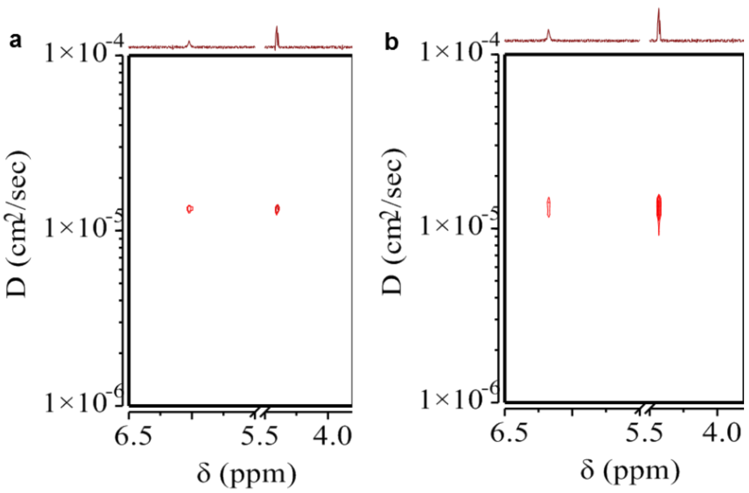

Supplementary Figure 22. DOSY spectra showing the diffusion coefficients of BLG-NCA in CD_2Cl_2. **a** in the absence of 18-C-6 and **b** in the presence of 18-C-6.

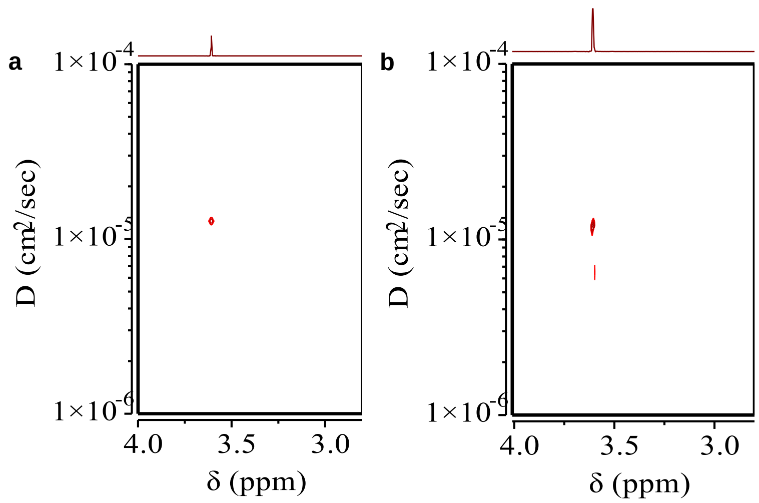

Supplementary Figure 23. DOSY spectra showing the diffusion coefficients of 18-C-6 in CD_2Cl_2. **a** in the absence of BLG-NCA and **b** in the presence of BLG-NCA.

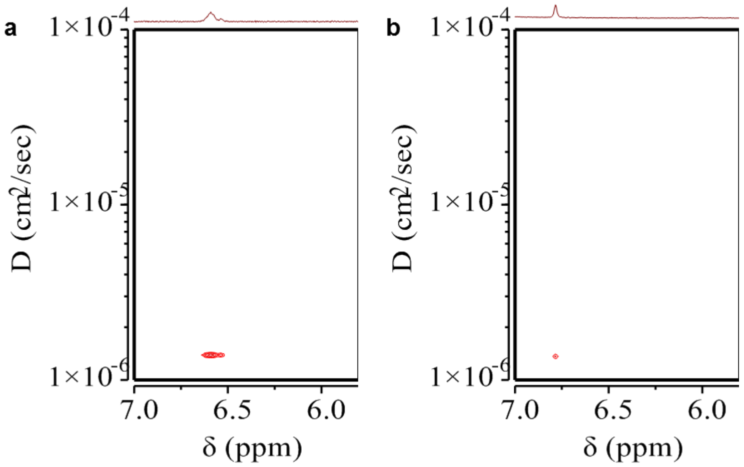

Supplementary Figure 24. DOSY spectra showing the diffusion coefficients of BLG-NCA in $CDCl_3$. **a** before mixing with PBLG-NH_2/18-C-6 and **b** after mixing with PBLG-NH_2/18-C-6.

Supplementary References

1. Baumgartner, R., Fu, H., Song, Z., Lin, Y. & Cheng, J. Cooperative polymerization of α-helices induced by macromolecular architecture. *Nat. Chem.* **9,** 614-622 (2017).
2. Zhang, R., Zheng, N., Song, Z., Yin, L. & Cheng, J. The effect of side-chain functionality and hydrophobicity on the gene delivery capabilities of cationic helical polypeptides. *Biomaterials* **35,** 3443-3454 (2014).
3. Song, Z. et al. Enzyme-mimetic self-catalyzed polymerization of polypeptide helices. *Nat. Commun.* **10,** 5470 (2019).
4. Habraken, G. J. M., Peeters, M., Dietz, C. H. J. T., Koning, C. E. & Heise, A. How controlled and versatile is *N*-carboxy anhydride (NCA) polymerization at 0 °C? Effect of temperature on homo-, block- and graft (co)polymerization. *Polym. Chem.* **1,** 514-524 (2010).
5. Zelzer, M. & Heise, A. Determination of copolymerisation characteristics in the *N*-carboxy anhydride polymerisation of two amino acids. *Polym. Chem.* **4,** 3896-3904 (2013).
6. Song, Z. et al. Synthesis of polypeptides via bioinspired polymerization of in situ purified *N*-carboxyanhydrides. *Proc. Natl. Acad. Sci. U. S. A.* **116,** 10658-10663 (2019).
7. Stejskal, E. O. & Tanner, J. E. Spin diffusion measurements: spin echoes in the presence of a time-dependent field gradient. *J. Chem. Phys.* **42,** 288-292 (1965).
8. Stejskal, E. O. Use of spin echoes in a pulsed magnetic-field gradient to study anisotropic, restricted diffusion and flow. *J. Chem. Phys.* **43,** 3597-3603 (1965).
9. Li, X., Li, Y., Yang, W. P., Chen, Y. Y. & Gong, S. L. Different metal-ion-induced dimeric self-assembling cavities based on thiacalix[4]benzocrown-4 isomers. *Dalton Trans.* **40,** 367-376 (2011).
10. Le, V. H., Yanney, M., McGuire, M., Sygula, A. & Lewis, E. A. Thermodynamics of host-guest interactions between fullerenes and a buckycatcher. *J. Phys. Chem. B* **118,** 11956-11964 (2014).
11. Yao, H. et al. Molecular recognition of hydrophilic molecules in water by combining the hydrophobic effect with hydrogen bonding. *J. Am. Chem. Soc.* **140,** 13466-13477 (2018).
12. Hirose, K. A practical guide for the determination of binding constants. *J. Incl. Phenom. Macro.* **39,** 193-209 (2001).
13. Malde, A. K. et al. An automated force field topology builder (ATB) and repository: version 1.0. *J. Chem. Theory Comput.* **7,** 4026-4037 (2011).
14. Becke, A. D. Density-functional thermochemistry. III. The role of exact exchange. *J. Chem. Phys.* **98,** 5648-5652 (1993).
15. Miertuš, S., Scrocco, E. & Tomasi, J. Electrostatic interaction of a solute with a continuum. A direct utilizaion of AB initio molecular potentials for the prevision of solvent effects. *Chem. Phys.* **55,** 117-129 (1981).
16. Singh, U. C. & Kollman, P. A. An approach to computing electrostatic charges for molecules. *J. Comput. Chem.* **5,** 129-145 (1984).
17. Schmid, N. et al. Definition and testing of the GROMOS force-field versions 54A7 and 54B7. *Eur. Biophys. J.* **40,** 843-856 (2011).

18. Abraham, M. J. et al. GROMACS: high performance molecular simulations through multi-level parallelism from laptops to supercomputers. *SoftwareX* **1-2,** 19-25 (2015).
19. Allen, M. P. & Tildesley, D. J. *Computer Simulation of Liquids*. (Clarendon Press, New York, 1989).
20. Essmann, U. et al. A smooth particle mesh Ewald method. *J. Chem. Phys.* **103,** 8577-8593 (1995).
21. Hess, B., Bekker, H., Berendsen, H. J. C. & Fraaije, J. G. E. M. LINCS: A linear constraint solver for molecular simulations. *J. Comput. Chem.* **18,** 1463-1472 (1997).
22. Nosé, S. A unified formulation of the constant temperature molecular dynamics methods. *J. Chem. Phys.* **81,** 511-519 (1984).
23. Parrinello, M. & Rahman, A. Polymorphic transitions in single crystals: a new molecular dynamics method. *J. Appl. Phys.* **52,** 7182-7190 (1981).
24. Hockney, R. W. & Eastwood, J. W. *Computer Simulation Using Particles*. (CRC Press, Bristol, 1988).
25. Humphrey, W., Dalke, A. & Schulten, K. VMD: visual molecular dynamics. *J. Mol. Graph.* **14,** 33-38 (1996).
26. Gowers, R. J. et al. MDAnalysis: a Python package for the rapid analysis of molecular dynamics simulations. In: *Proceedings of the 15th Python in Science Conference* (eds Benthall S, Rostrup S) (2016).
27. Michaud-Agrawal, N., Denning, E. J., Woolf, T. B. & Beckstein, O. MDAnalysis: a toolkit for the analysis of molecular dynamics simulations. *J. Comput. Chem.* **32,** 2319-2327 (2011).
