# Supplementary material for: Accelerated polymerization of N-carboxyanhydrides catalyzed by crown ether
Source: Nat Commun. 2021 Feb 2;12:732. doi: 10.1038/s41467-020-20724-w (PMC7854670; doi:10.1038/s41467-020-20724-w)
Supplement: Supplementary file 3 — Description of Additional Supplementary Files [file 41467_2020_20724_MOESM3_ESM.pdf]

Description of Additional Supplementary Files

File Name: Supplementary Movie 1
Description: Movie illustrating the rapid polymerization of BLG-NCA in the presence of 18-C-6, with the vigorous evolution of CO_2 bubbles. $[M]_0/[I]_0 = 100$, $[I]_0 = [CE]_0 = 4$ mM.

File Name: Supplementary Movie 2
Description: Molecular dynamics simulation of 30 mM DBLG and 3 mM BLG-NCA in DCM. Simulations of 105 DBLG, 11 BLG-NCA, and 51,920 DCM molecules were conducted at 298 K and 1 bar over the course of 10 ns. Snapshots are visualized every 10 ps. DCM solvent is not visualized for clarity. Movie is constructed using Visual Molecular Dynamics[25].

File Name: Supplementary Movie 3
Description: Molecular dynamics simulation of 30 mM DBLG, 3 mM BLG-NCA, and 3 mM 18-C-6 in DCM. Simulations of 105 DBLG, 11 BLG-NCA, 11 18-C-6, and 51,839 DCM molecules were conducted at 298 K and 1 bar over the course of 10 ns. Snapshots are visualized every 10 ps. DCM solvent is not visualized for clarity. Movie is constructed using Visual Molecular Dynamics[25].
